# A Coding Sequence-Embedded Principle Governs Translational Reading Frame Fidelity

**DOI:** 10.1155/2018/7089174

**Published:** 2018-09-20

**Authors:** Ji Wan, Xiangwei Gao, Yuanhui Mao, Xingqian Zhang, Shu-Bing Qian

**Affiliations:** ^1^Division of Nutritional Sciences, Cornell University, Ithaca, NY 14853, USA; ^2^Graduate Programs in Genetics Genomics and Development, Biochemistry Molecular and Cellular Biology, Cornell University, Ithaca, NY 14853, USA

## Abstract

Upon initiation at a start codon, the ribosome must maintain the correct reading frame for hundreds of codons in order to produce functional proteins. While some sequence elements are able to trigger programmed ribosomal frameshifting (PRF), very little is known about how the ribosome normally prevents spontaneous frameshift errors that can have dire consequences if uncorrected. Using high resolution ribosome profiling data sets, we discovered that the translating ribosome uses the 3′ end of 18S rRNA to scan the AUG-like codons after the decoding process. The postdecoding mRNA:rRNA interaction not only contributes to predominant translational pausing, but also provides a retrospective mechanism to safeguard the ribosome in the correct reading frame. Partially eliminating the AUG-like “sticky” codons in the reporter message leads to increased +1 frameshift errors. Remarkably, mutating the highly conserved CAU triplet of 18S rRNA globally changes the codon “stickiness”. Further supporting the role of “sticky” sequences in reading frame maintenance, the codon composition of open reading frames is highly optimized across eukaryotic genomes. These results suggest an important layer of information embedded within the protein-coding sequences that instructs the ribosome to ensure reading frame fidelity during translation.

## 1. Introduction

The importance of the translation machinery and its fidelity have been apparent since the discovery of the genetic code [1]. During translation, the ribosome reads nucleotide triplets in a successive, nonoverlapping fashion. For a given coding sequence, it is the start codon (most likely AUG) that establishes the reading frame. Since there is no punctuation between codons for successive amino acid residues, all three reading frames give rise to different codon sequences. However, only one reading frame primarily serves as the coding template for functional proteins because a high frequency of stop signals exists in alternative frames [2]. This feature represents a strong evolutionary optimization of codon sequences that minimizes the consequence of frameshift errors [3]. Unlike missense errors, which are not necessarily destructive to proteins, frameshift errors are deleterious as premature termination often leads to nonfunctional translational products. Therefore, the ribosome must keep the correct reading frame for hundreds to thousands of codons during translation to ensure proper protein production.

Previous attempts to investigate the mechanisms underlying reading frame maintenance have focused on the interaction between mRNA and tRNA at the decoding center [4]. Although codon:anticodon pairing is central to decoding accuracy, there is an extensive network of interaction between mRNA, rRNA, and ribosomal proteins [5]. The stepwise, codon by codon, progression of the ribosome along the mRNA from the 5′ to 3′ direction is primarily driven by translocation [6]. During this step, the ribosome undergoes massive conformational rearrangement, allowing movement of P- and A-site tRNAs into the E- and P-sites, respectively [7–10]. It is thought that the coordinated rotation of large and small subunits ensures one codon precision in the movement of mRNA and the attached tRNAs after peptidyl transfer [11]. However, tremendous flexibility exists. In bacteria, while elongation factor G (EF-G) mediates one codon forward movement of mRNA:tRNA complexes, elongation factor 4 (EF4) catalyzes back-translocation [12]. Lack of EF4 has been shown to reduce translation fidelity under stress conditions [13], suggesting that translocation is error-prone.

While ribosomes must maintain the correct reading frame in order to produce functional proteins, some* cis*-acting signals located in mRNAs are able to trigger programmed ribosomal frameshifting (PRF) [14]. Such recoding events have been exploited by viruses to expand the genetic coding capacity within their limited genome size [15, 16]. The typical signals for –1 PRF include a slippery sequence, a downstream secondary structure (usually a pseudoknot), and a short spacer in between. With an increasing amount of PRF signals identified, it is clear that the ribosome bears tremendous flexibility in decoding mRNAs. The rich communication between the ribosome and the message is thus equally, if not more, important as codon:anticodon pairing in maintaining translation fidelity. However, our understanding of individual PRF cases provides limited insights on how the ribosome normally maintains the correct reading frame [17–20]. The fundamental question centers on how the ribosome is able to achieve remarkable precision for the majority of mRNA coding sequences while permitting off-track in certain regions.

Here we set out to investigate mechanistic features of coding sequences in translational reading frame fidelity. Using high resolution ribosome profiling data sets obtained from eukaryotic cells, we uncovered universal “sticky” sequences within the coding region that retains the ribosome by pairing with 18S rRNA. Analogous to the internal Shine-Dalgarno (SD) sequences in prokaryotic mRNAs [21], the eukaryotic “sticky” sequences contribute to pervasive translational pausing. Importantly, the interaction between the mRNA “sticky” sequence and the 3′ end of 18S rRNA offers a gripping mechanism after decoding to guide the ribosome in the correct reading frame during translation. In addition, we found that mRNA coding sequences are subject to multilevel optimization during evolution to minimize frameshift errors. Our work illustrates new layers of information embedded within protein-coding sequences that extends our understanding of the genetic code.

## 2. Results

### 2.1. Measuring Spontaneous Frameshift Rates during Translation

**Figure 1:**
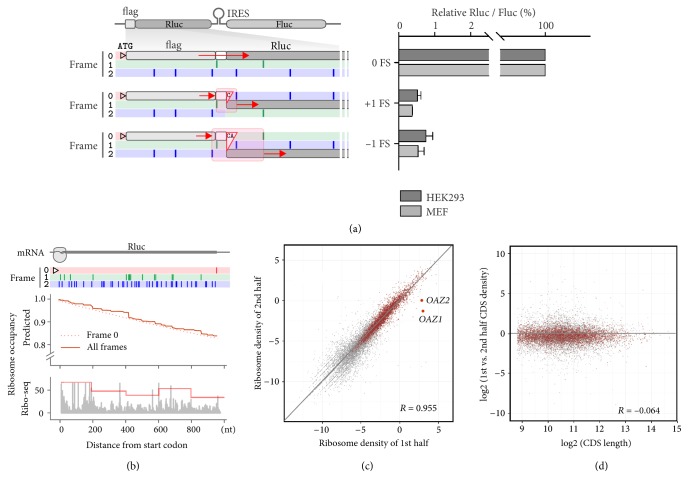
**Discrepancy between experimental FS rates and ribosomal occupancy. (a)** Schematic of FS reporters based on a bicistronic expression construct containing CrPV IRES. For the enlarged region of flag-Rluc, all three reading frames are color coded with stop sites shown as perpendicular lines. The FS windows are highlighted in red shade. The right panel shows the relative Rluc activities in transfected cells. Data are shown as means ± SEM, n = 3.** (b)** The top panel shows the gene structure of Rluc with all three reading frames and stop sites color coded. The middle panel shows the predicted ribosome occupancy along the Rluc coding region based on the +1 FS rate of 0.03% and –1 FS of 0.02%. The bottom panel shows the density of RPFs mapped to the Rluc coding region in transfected HEK293 cells. The line plot represents average ribosome density within a window of 200 nt.** (c)** Using ribosome profiling data in HEK293 cells, average ribosome density is plotted for the first and second half of each ORF. Genes with no less than 50 read counts in at least half of the CDS are shown in dark red.** (d)** Using ribosome profiling data in HEK293 cells, the ratio of average ribosome density between first and second half of each ORF is plotted against the ORF size. Genes with no less than 50 read counts in at least half of the CDS are shown in dark red.

To understand how ribosomes normally maintain translational reading frame fidelity, it is necessary to determine the spontaneous frameshift (FS) rate under physiological conditions. We adopted a luciferase-based bicistronic reporter assay, in which both the* Renilla* luciferase (Rluc) and the Firefly luciferase (Fluc) are synthesized from the same mRNA (Figure 1(a)) [22]. A flag-tag was added at the 5′ end of the Rluc coding region after the start codon and the full length flag-Rluc fusion protein serves as control for in-frame translation. For the +1 FS reporter, we inserted a single cytosine (C) between flag and Rluc, thereby placing the entire Rluc coding region into frame 1. Since no AUG start codons are present in frame 1 before the Rluc coding region, any detectable Rluc activities are derived from the +1 FS event during flag synthesis. To eliminate Fluc products via leaky scanning, we conducted anti-Flag immunoprecipitation in parallel. Notably, only after the FS events taking place within a window delimited by the nearest stop codons flanking the insertion site, can the shifted ribosome reach frame 1 coding region of Rluc (Figure 1(a), highlighted region). Compared to the in-frame control, we observed a reproducible +1 FS rate of 0.52% in HEK293 cells and 0.37% in MEF cells (Figure 1(a), right panel). After adjustment by the size of the FS window, the average +1 FS rate per codon in HEK293 and MEF cells is 0.04% and 0.03%, respectively. We next constructed a –1 FS reporter by inserting two additional bases (CA) between flag and Rluc. Once the ribosome skips two bases or slips back one base within the FS window, it will reach frame 2 Rluc coding region for translation. Compared to the +1 FS reporter, we observed a relatively lower –1 FS rate of 0.03% in HEK293 and 0.02% in MEF after adjustment by the size of the FS window (Figure 1(a)). This value is in the similar range of spontaneous FS rates reported previously [23].

The seemingly low FS rates measured from the reporter assay could have striking effects on ribosome engagement if the FS rate remains constant over the entire coding region. Even with the lower bound FS rate (0.03% for +1 FS and 0.02% for –1 FS), the theoretical exponential decay predicts that nearly 20% of ribosomes will be derailed from the mRNA before reaching the stop codon of Rluc (Figure 1(b)). To experimentally determine the pattern of ribosome occupancy along the Rluc coding region, we conducted ribosome profiling in HEK293 cells transfected with the control bicistronic plasmid [24, 25]. In spite of the nonuniform read distribution, the average trend of ribosome occupancy along the Rluc coding region does not parallel with the predicted exponential decay (Figure 1(b), bottom panel).

We next assessed global ribosome drop-off across endogenous transcripts by comparing the average ribosome density for the first and second half of each coding region (CDS) in HEK293 cells (Figure 1(c)) [26]. The strong correlation (*R* = 0.955) between the two regions indicates that there is minimal drop-off in ribosome occupancy for the vast majority of genes ([Sec supplementary-material-1]). If spontaneous FS errors occur in a continuous manner, the overall ribosome drop-off is expected to increase as function of CDS length. However, the ratio of ribosome density between the first and second half of CDS largely maintains irrespective of the CDS size (Figure 1(d)). The same pattern also holds true in MEF cells ([Sec supplementary-material-1] and [Sec supplementary-material-1]). Interestingly, a few genes that display large drop-off are known examples of PRF. For instance, both* OAZ1* and* OAZ2* undergo controllable +1 PRF, allowing synthesis of ornithine decarboxylase (ODC) antizymes [27] (Figure 1(c) and [Sec supplementary-material-1]).

### 2.2. Effects of RPF Codon Identity on Reading Frame Fidelity

The discrepancy between the experimental FS rates and ribosomal occupancy suggests that the overall frameshift consequence cannot be extrapolated from the local region in a linear manner. To assess the natural reading frame accuracy of individual ribosomal steps, we took advantage of ribosome profiling data sets that permit determination of ribosome positions at a subcodon resolution [28]. Similar to previous reports [29], the overall ribosome-protected fragments (RPFs) show a strong 3-nucleotide periodicity with approximately 70% of reads mapped to frame 0 ([Sec supplementary-material-1]). The remaining out-of-frame reads could be due to technical artifacts. For instance, nuclease overdigestion or incomplete digestion will shift the resultant RPF from frame 0 to frame 1 or 2, respectively. Despite these inevitable variations, specific FS events occurring at particular regions could be discernable by altered phasing [30, 31]. We computed the in-frame rate (IFR) for footprints mapped to individual codon positions by calculating the percentage of RPFs aligned to frame 0. The IFR value represents, on a relative scale, how accurately the ribosome is positioned when a particular codon is encountered.

**Figure 2:**
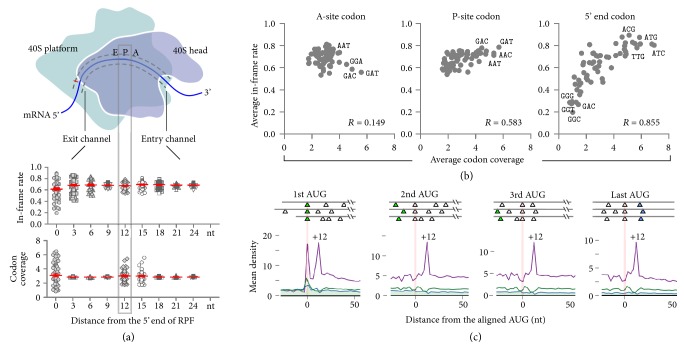
**The 5**′** end codon identity of footprints influences read abundance and reading frame fidelity. (a)** The top panel depicts the mRNA tunnel on the small ribosome subunit. Red arrow indicates the 5′ end of RPF generated by Ribo-seq. RPFs from HEK293 cells are stratified by the identity of codons at different positions of footprints. Their corresponding IFR values (middle panel) and abundance (bottom panel) are group plotted. Red line, mean ± SD.** (b)** RPFs are grouped based on the codon identity at A-site, P-site, and 5′ end of footprints. All 61 sense codons are plotted by the average codon coverage score of RPFs (x-axis) against the average in-frame rate (y-axis).** (c)** Metagene analysis of RPFs obtained from HEK293 cells. Transcripts are aligned at the annotated start codon (1st AUG, green triangle) or internal AUG codons (blank triangle). The average read density at each nucleotide position is plotted using the P-site of RPFs stratified by reading frames (magenta, frame 0; blue, frame 1; green, frame 2). The aligned codon is highlighted, which corresponds to the 5′ end of footprints when the P-site is at the +12 codon position.

A typical ribosome footprint covers 10 or so codons (Figure 2(a)). If the codon identity at each position does not contribute to reading frame maintenance, we expect to see uniform IFR values among the 61 sense codons. Using the ribosome profiling data from HEK293 cells, we computed the average IFR for RPFs with specific codons at individual positions of the footprint. As expected, RPFs with different 3′ end codons show little variation of IFR (< 1.2-fold, Figure 2(a)), because those codons are not yet being decoded. For RPFs grouped by P-site or A-site codons, we observed a modest variation of IFR (~1.5-fold). To our surprise, when the 5′ end codon identity is considered, there is a substantial variation of IFR (> 4.5-fold) among the 61 sense codons ranging from 0.2 to 0.9 (Figure 2(a)). This feature is independent of the RPF length because we observed a nearly identical pattern for RPFs with a fixed length of 28 nt ([Sec supplementary-material-1]). Interestingly, for footprints with IFR values above the average, their 5′ end codons mostly belong to AUG-like triplets (i.e., codons that differ from AUG by a single nucleotide) (Figure 2(b) and [Sec supplementary-material-1]). This salient feature is highly reproducible in biological replicates and in different cell lines ([Sec supplementary-material-1]).

We next examined whether the codon identity affects the relative abundance of RPFs. The codon-specific RPF abundance is determined by calculating average codon coverage, which represents how frequently we observe ribosomes when that codon appears at specific positions of the footprint. Previous studies have focused on codon identity at the decoding center and the effect on ribosomal pausing [32]. Indeed, both the A-site and P-site codon identities influence the RPF abundance (Figure 2(a), bottom panel). Intriguingly, RPFs grouped by 5′ end codons display the greatest variation of abundance with prominent pausing that appeared for footprints starting with AUG-like codons (Figures 2(a) and 2(b)). In contrast to A-site or P-site codons that exhibit only weak correlation between the reading frame accuracy and the ribosome dwell time, the 5′ end codon identity of RPFs influences both footprint abundance and reading frame fidelity in a coordinated manner (Spearman's* R* = 0.855, Figure 2(b)). This result suggests that the 5′ end codon-associated ribosomal pausing is coupled with reading frame fidelity.

### 2.3. Uncovering Biological Meanings of RPF 5′ End Codon Preference from Sequencing Bias

We previously attributed the elevated ribosome density at the +12 nt position after the AUG start codon to the peptide tunnel within the ribosome [33]. However, the increased read peak also emerges at the same position after internal AUG codons, including the last AUG (Figure 2(c)). Therefore, this feature is unlikely due to the initiation event. Indeed, we observed the same 5′ end codon preference after excluding the first 30 nt of CDS ([Sec supplementary-material-1]). For many deep sequencing results, the 5′ end of reads is subject to technical bias [34]. The commonly found A/T enrichment at the 5′ end of reads in Ribo-seq and RNA-seq is possibly due to unequal ligation during library construction [35]. Therefore, it is possible that the overrepresentation of AUG codon at the 5′ end of RPFs is a result of sequencing bias. To uncover true footprint features masked by sequencing bias, we took several independent approaches to characterizing RPFs. First, we compared ribosome profiling and RNA-seq data sets generated using the same protocol from the same cells. Indeed, RNA-seq reads exhibit an obvious A/T enrichment for the first nucleotide ([Sec supplementary-material-1]). However, our Ribo-seq reads mainly start with A, but not T. Despite the common A preference for the 5′ end nucleotide, RNA-seq reads exhibit poor correlation between read abundance and IFR for their 5′ end codons. Therefore, although some ribosome footprints could be inflated as a result of sequencing bias, they must originally dwell in the right reading frame. Second, the high IFR values associated with certain RPFs could also be seen in the absence of cycloheximide (CHX) ([Sec supplementary-material-1]), excluding the possible side effect of translation inhibitors by altering ribosomal conformations [36]. Third, we stratified RPFs based on read length and found the consistent pattern of IFR and codon coverage ([Sec supplementary-material-1]). Thus, the 5′ end bears reliable information in inferring ribosome positions and reading frames. Fourth, we reanalyzed a series of ribosome profiling data sets published previously by different research groups [37]. Despite the varying data quality and using different species, RPFs with certain 5′ end codons are well separated in terms of IFR values ([Sec supplementary-material-1]). In particular, the AUG codon clearly stands out with prominent read density and reading frame fidelity.

Since Ribo-seq collects footprints from cell lysates, it is possible that footprints starting with certain codons are better protected by ribosomes* in vitro*. To confirm whether the 5′ end codon preference also exists* in vivo*, we took advantage of the data set generated by 5P-seq [38]. 5P-seq captures naturally occurring 5′ end of footprints as a result of mRNA decay ([Sec supplementary-material-1]). Since 5P-seq also involves the ligation step, a similar A/T bias is found at the 5′ end of 5P-seq reads ([Sec supplementary-material-1]). Notably, 5P-seq revealed a shifted 5′ end position compared to Ribo-seq ([Sec supplementary-material-1]). Only after a 4 nt adjustment, could we observe the prominence of AUG in both IFR and read abundance ([Sec supplementary-material-1]). Since the virtual 5′ end is not part of the sequencing reads in 5P-seq, this result affirms that the 5′ end codon preference observed in ribosome footprints is not a result of experimental aberrations. Additionally, we observed a positive correlation between Ribo-seq and 5P-seq results in both 5′ end codon coverage and in-frame rate ([Sec supplementary-material-1]).

### 2.4. mRNA:rRNA Pairing Influences Ribosome Pausing and Reading Frame Fidelity

**Figure 3:**
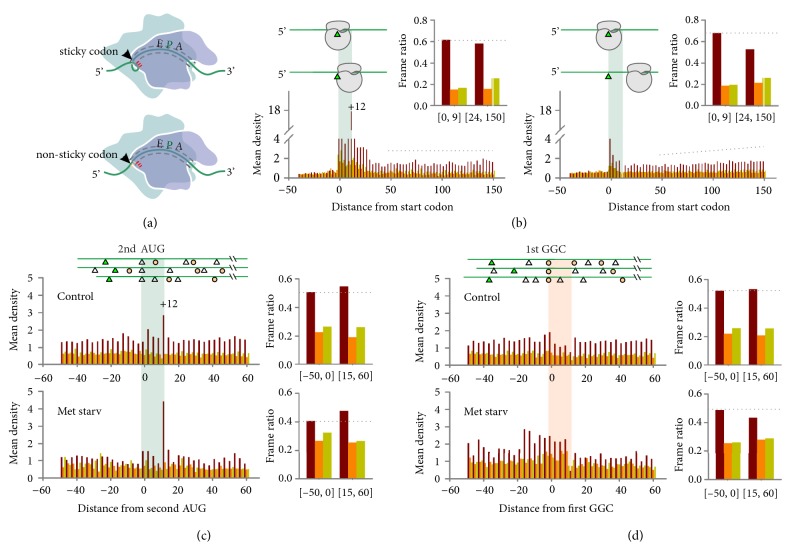
**“Sticky” codon-associated ribosomal pausing facilitates downstream reading frame fidelity. (a)** Proposed mRNA:rRNA interaction in eukaryotic translation. When the “sticky” codon reaches the exit site of the mRNA tunnel, the mRNA likely loops back towards the ribosome (top panel). In the absence of “sticky” codons, the mRNA takes on an extended conformation at the exit site (bottom panel).** (b)** Aggregation plots using RPFs derived from transcripts with (left) or without (right) pausing at the +12 nt position. Reading frames are color coded. The inserted bar graph shows averaged frame ratio in different regions as indicated.** (c)** Aggregation plots of transcripts aligned with the first AUG codon (white triangle) after the start codon (green triangle). RPFs were derived from HEK293 cells with (bottom) or without (top) amino acid starvation. Reading frames are color coded. The right side bar graphs show averaged frame ratio in different regions as indicated.** (d)** Aggregation plots of transcripts aligned with the first GGC codon (orange circle) after the start codon (green triangle). RPFs were derived from HEK293 cells with (bottom) or without (top) amino acid starvation. Reading frames are color coded. The right side bar graphs show averaged frame ratio in different regions as indicated.

How does the 5′ end codon identity of footprints influence the residence time of ribosomes? A previous study using UV crosslinking reported extensive interaction between the small subunit 40S and the bound mRNA [39]. In particular, the 3′-terminal of 18S rRNA cross-linked to mRNA positions of –8 to –11 nt (relative to the +1 of the P-site), which corresponds to the 5′ end of ribosome footprints. Given the highly conserved 3′-terminal sequence of 18S rRNA among all eukaryotic species ([Sec supplementary-material-1]), we hypothesize that the AUG-like codons of mRNAs interact with the 3′-proximal CAU triplet of 18S rRNA, thereby retaining the translating ribosome. Supporting this notion, an elevated read density appears unanimously at the +12 nt position following the start site as well as internal AUG codons (Figure 2(c)). Given the 12 nt offset between the mapped P-site and the 5′ end, it is likely that the ribosome pauses whenever the AUG codon reaches the mRNA exit site of the ribosome after decoding. Notably, the same feature is also evident for other AUG-like codons, such as AUC, but not non-AUG codons like GUC ([Sec supplementary-material-1]). We termed these AUG-like codons of mRNA as “sticky” sequences as they tend to retain the ribosome during elongation (Figure 3(a)).

The feature of “sticky” codons in eukaryotic mRNAs is analogous to the Shine-Dalgarno (SD) sequence found in bacterial messages. It is expected that the first event of such interaction occurs when the ribosome decodes the 5th codon, where the tail of 18S rRNA reaches the start codon AUG (Figure 3(b)). Intriguingly, we found that the +12 nt ribosomal pausing was far from uniform, suggesting that other factors may influence the putative mRNA:rRNA interaction, such as mRNA folding, modification, or ribosome stacking. We classified all the transcripts into two groups depending on whether the +12 nt peak is above or below the average ribosome density. For messages without +12 nt ribosomal pausing, the downstream region showed a surprisingly lower average IFR value than the first 3 codons. Notably, the downstream IFR was gradually restored to the average level (Figure 3(b), right panel). However, it is possible that these transcripts contain overlapping out-of-frame ORFs in the 5′-proximal region. We next compared the regional IFR before and after internal “sticky” or “non-sticky” codons. When transcripts were aligned to the second AUG (first internal AUG) codon, we observed a modest increase of IFR values averaged from the downstream region relative to the upstream (Figure 3(c), top panel). This feature was not seen when transcripts were aligned to the first GGC codon. We speculate that the putative mRNA:rRNA interaction during elongation helps maintain the reading frame fidelity in downstream coding regions.

The seemingly modest effect of a single AUG codon (7.6%) in maintaining the reading frame of downstream regions could be substantial when multiple internal AUG-like codons are considered. To demonstrate whether the internal mRNA:rRNA pairing could potentially adjust out-of-frame translation, we attempted to induce out-of-frame translation by incubating HEK293 cells in a culture medium devoid of methionine. As expected, methionine starvation potently suppressed AUG-initiated translation and gave rise to footprints with mixed reading frames ([Sec supplementary-material-1] and [Sec supplementary-material-1]), presumably due to non-AUG translation of upstream and downstream ORFs. To our surprise, in the absence of methionine, there was an outstanding ribosome pausing at the +12 nt position following the start codon as well as internal AUG codons (Figure 3(c) and [Sec supplementary-material-1]). Despite the poor IFR in regions before the internal AUG, a prominent ribosome pause at this “sticky” codon resulted in a much improved IFR with an increase of 17% (Figure 3(c), bottom panel). By contrast, when a typical “non-sticky” codon GGC was chosen to align the messages, we observed a decreased IFR value for the downstream region (Figure 3(d)). Collectively, these results support the notion that mRNA:rRNA pairing during translation helps retain the ribosome in the reading frame originally set by the AUG start codon.

### 2.5. mRNA:rRNA Pairing Exhibits Reading Frame-Specific Effects

**Figure 4:**
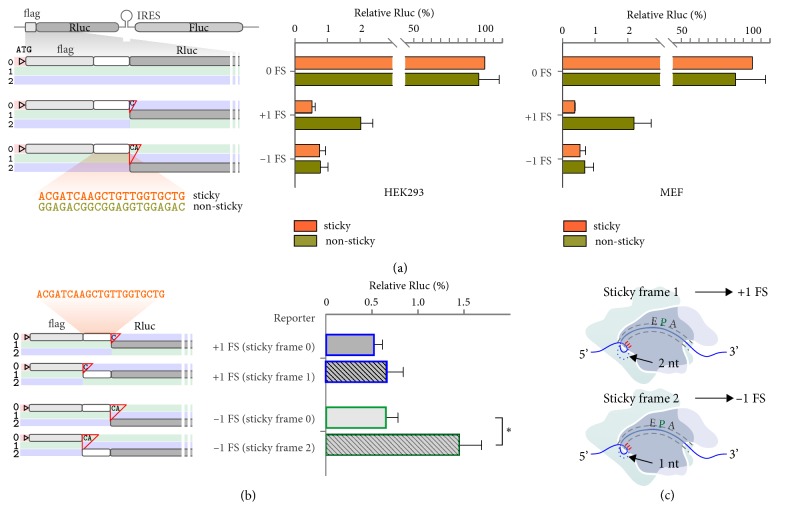
**mRNA:rRNA pairing exhibits reading frame-specific effects. (a)** The left panel depicts schematic of FS reporters with “sticky” or “non-sticky” inserts in the frame 0 between flag and Rluc. The right panels show the relative Rluc activities in transfected HEK293 and MEF cells. Data are shown as means ± SEM, n = 3.** (b)** The left panel depicts schematic of FS reporters with “sticky” inserts in different frames between flag and Rluc. The right panels show the relative Rluc activities in transfected cells. Data are shown as means ± SEM, n = 3. *∗ p* <0.01,* t*-test.** (c)** Schematic of mRNA:rRNA pairing in the small ribosome subunit when the “sticky” codon is present in frame 1 (top panel) or frame 2 (bottom panel). Inclusion of extra 1 nt in the mRNA loop is more favorable and likely results in –1 FS.

To experimentally dissect the differential effects of “sticky” versus “non-sticky” sequences on reading frame fidelity, we employed +1 and –1 FS reporters by introducing a 21-mer of extra sequences between flag and Rluc but before the FS insertion site (Figure 4(a)). Both “sticky” and “non-sticky” sequences were carefully chosen to avoid AUG and stop codons in all three reading frames. By normalizing to the in-frame control, the Rluc levels in transfected cells represent the extent of corresponding FS events. Notably, the insertion of different amino acids had little effect on Rluc activities, although the presence of “non-sticky” sequence slightly lowered the Rluc level in the in-frame control (Figure 4(a), right panel). However, for the +1 FS reporter containing the “non-sticky” sequence, the Rluc level was about 4-fold higher than the “sticky” counterpart in both HEK293 and MEF cells. Intriguingly, the marked difference seen in the +1 FS reporter was not observable in the –1 FS reporter. To exclude possible crypt splicing events or hidden promoters generated after insertion of extra sequences, we repeated these experiments by transfection of cells using capped mRNAs synthesized* in vitro*. We obtained nearly identical results in both HEK293 and MEF cells ([Sec supplementary-material-1]). Our data indicate that the lack of “sticky” codons along the coding region triggers high frequency of +1 FS events, but not –1 FS. This is in line with the model that mRNA:rRNA pairing serves as a “brake” at the outside of the mRNA tunnel to prevent overmovement of the message during translocation.

The AUG-like codons do not exclusively exist in frame 0. It is unclear whether these “sticky” sequences would promote frameshifting once they are placed in different reading frames. To address this question, we constructed FS reporters by inserting the “sticky” sequence into different reading frames preceding the Rluc coding region (Figure 4(b)). Constructs with frame 0 “sticky” insert were used as control. For the +1 FS reporter, we observed negligible difference of Rluc levels in transfected cells regardless of the frames the “sticky” sequence residing in Figure 4(b). However, for the –1 FS reporter, we observed greater than 2-fold Rluc activities in transfected cells when the “sticky” insert was relocated from frame 0 to frame 2. Once again, mRNA transfection gave rise to the similar results, confirming the reading frame-specific effects of “sticky” sequence in driving frameshifting ([Sec supplementary-material-1]). The finding that frame 2 “sticky” sequence is easier to induce FS than the same sequence in frame 1 is surprising but consistent with the “gripping” model of mRNA movement imposed by mRNA:rRNA pairing (Figure 4(c)). When the AUG-like codons are present in frame 1, mRNA:rRNA pairing is less favorable than the one bearing “sticky” codons in frame 2 ([Sec supplementary-material-1]). To resolve the extra nucleotide in the mRNA loop, it is relatively easier to push back 1 nt upon translocation, resulting in –1 FS (Figure 4(c)).

### 2.6. Mutant 18S rRNA Alters Global Codon “Stickiness”

**Figure 5:**
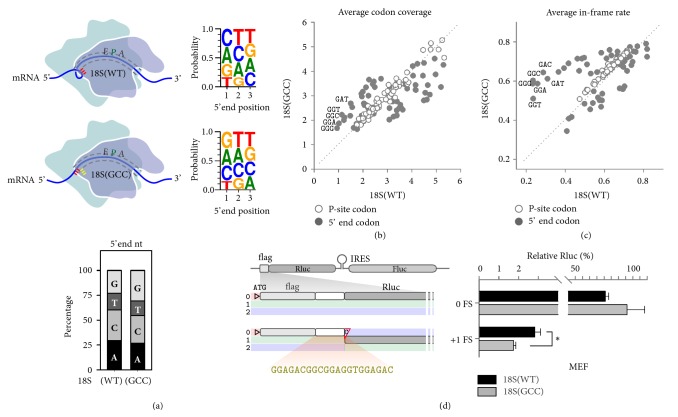
**Mutant 18S rRNA alters global codon “stickiness”. (a)** Schematic of mRNA:rRNA pairing in the small ribosome subunit bearing wild type (CAU triplet shown as red) or mutant 18S rRNA (GCC triplet as yellow). Sequence logo shows the probability of individual nucleotides at 5′ end positions of RPFs derived from MEF cells expressing 18S(WT) or 18S(GCC).** (b)** All 61 sense codons are plotted by the average codon coverage score of RPFs with specified P-site codons (white circle) or 5′ end codons (grey circle) in HEK293 cells expressing wild type (x-axis) or mutant (y-axis) 18S rRNA.** (c)** All 61 sense codons are plotted by the average IFR of RPFs with specified P-site codons (white circle) or 5′ end codons (grey circle) in HEK293 cells expressing wild type (x-axis) or mutant (y-axis) 18S rRNA.** (d)** The left panel depicts schematic of FS reporters with a specific sequence insert in frame 0 between flag and Rluc. The right panels show the relative Rluc activities in transfected cells. Data are shown as means ± SEM, n = 3. *∗ P* <0.01,* t*-test.

To further demonstrate the unexpected role of mRNA:rRNA pairing in ribosomal pausing as well as reading frame maintenance, we attempted to evaluate the behavior of ribosomes bearing a 18S rRNA mutant with altered mRNA:rRNA interaction. The presence of hundreds of rDNA copies in cells prevents us from using genome editing tools like CRISPR/Cas9. Instead, we used a previously described 18S rRNA expression system that has been shown to be able to incorporate the exogenously expressed 18S rRNA into ~15% of 40S subunits in transfected cells [40]. To change the base pairing between 18S rRNA and mRNA, we mutated the 3′ terminal CAU triplet of 18S rRNA into GCC (Figure 5(a)). The resultant 18S(GCC) mutant is expected to establish new base pairing with mRNA codons like GGC, one of the typical “non-sticky” codons for wild type 18S rRNA. Therefore, global changes of codon “stickiness” in the presence of 18S(GCC) mutant will help us validate the critical role of mRNA:rRNA pairing in translation fidelity.

We conducted ribosome profiling of MEF cells transfected with either 18S(WT) or 18S(GCC) followed by in-depth RPF analysis. Interestingly, for RPFs mapped to mRNAs, the usually underrepresented nucleotide G now becomes dominant in cells expressing 18S(GCC) (Figure 5(a)). This result further suggests that the 5′ end sequence preference of RPFs does not entirely stem from technical artifacts. We examined the abundance of RPFs with specific codons at the P-site or the 5′ end ([Sec supplementary-material-1]). RPFs grouped by P-site codons exhibit little change of abundance in cells expressing 18S(GCC) (Figure 5(b)). Thus, the 3′ tail of 18S rRNA does not influence the residence time of ribosomes during decoding. However, when the footprint 5′ end codon identity was analyzed, RPFs starting with GGC-like codons showed an evident increase of abundance in cells expressing 18S(GCC). Metagene analysis of transcriptome aligned at specific codons revealed a clear switch of codon “stickiness” as judged by the +12 nt peak height between wild type and mutant 18S rRNA ([Sec supplementary-material-1]). These results indicate that the 18S rRNA mutant alters the relative “stickiness” of mRNA codons by reestablishing mRNA:rRNA pairing during translation.

We next evaluated the reading frame fidelity of ribosome footprints in cells expressing either 18S(WT) or 18S(GCC). For RPFs with different P-site codon identity, their IFR values were nearly identical in both transfected cells (Figure 5(c)). Only when the 5′ end codon identity was compiled, did certain codons exhibit skewed IFR values for their footprints. For RPFs with increased IFR in the presence of 18S(GCC), many of their 5′ end codons belong to GGC-like triplets. The relatively higher sensitivity of GGC-like codons to the 18S(GCC) mutant is likely owing to their low basal levels of IFR. Although only a small portion of 40S subunits were incorporated with 18S(GCC), the true effect of mutant 18S rRNA would be even greater.

Since insertion of “non-sticky” sequence into frame 0 triggered high frequency of +1 FS (Figure 4(a)), we predicted that overexpression of 18S(GCC) mutant should suppress +1 FS. This was indeed the case. We observed about 38% reduction of +1 FS in cells transfected with 18S(GCC) as compared to the 18S(WT) (Figure 5(d)). Intriguingly, the in-frame control bearing the “non-sticky” sequence showed an increased Rluc levels in the presence of 18S(GCC). Taken together, changing mRNA:rRNA interaction not only alters global “stickiness” of sequences, but also influences spontaneous frameshifting errors during translation.

### 2.7. “Sticky” Codons Exhibit Frame-Specific Distribution

**Figure 6:**
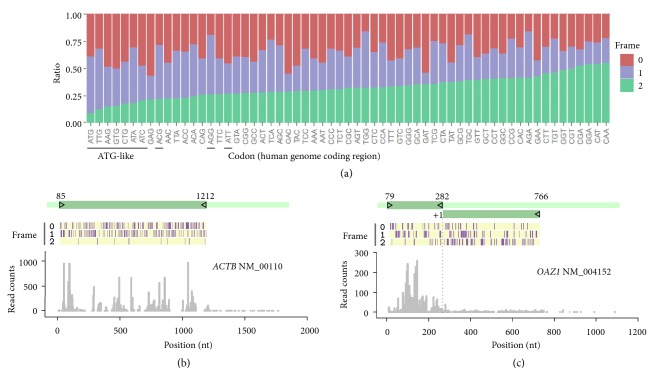
** “Sticky” codons exhibit frame-specific features. (a)** Relative ratio of 61 sense codons in three reading frames of coding regions within the human genome.** (b)** Distribution of “sticky” codons and CDS ribosome density on *β*-actin gene* ACTB*. Gene structure is shown above with all “sticky” codons represented as perpendicular lines in all three reading frames. Ribosome density across the CDS is shown below.** (c)** Distribution of “sticky” codons and CDS ribosome density on* OAZ1*. +1 PRF site is indicated by dotted line.

Up to this point, it is clear that the ribosome not only reads the message via the decoding center, but also scans the mRNA using the tail of 18S rRNA. The postdecoding mRNA:rRNA interaction plays an unexpected role in reading frame fidelity and exerts a surprising reading frame-specificity. For instance, mRNA:rRNA pairing only prevents +1 FS but not –1 FS (Figure 4(a)). In addition, the “sticky” sequence is rather safe in frame 1 because it does not induce +1 frameshifting errors (Figure 4(b)). Since “sticky” codons, when present in frame 2, promote –1 FS by potentially reengaging ribosomes, we reasoned that AUG-like codons must be avoided in frame 2 during evolution to minimize spontaneous FS errors. We computed the codon composition in different reading frames for the entire coding region of human genome (Figure 6(a)). Intriguingly, nearly all of the ATG-like codons are kept at minimal levels in frame 2 with the ATG itself the lowest. To exclude the possibility that the codon preference in frame 2 is a result of codon selection in frame 0, we randomized whole codons while maintaining the original amino acid sequence. Remarkably, ATG and GGC codons in frame 2 of randomized sequences are clearly deviated from the same codons in human CDS ([Sec supplementary-material-1]). Similar results were obtained after dinucleotide randomization. Therefore, the codon preference in frame 2 is not a consequence of codon bias that existed in frame 0. Additionally, the asymmetric distribution of “sticky” codons among reading frames is highly conserved across different eukaryotic genomes ([Sec supplementary-material-1]). These results clearly indicate that there is a universal trend of selection for low frequency of “sticky” codons in frame 2. As a typical example, the *β*-actin gene* ACTB* showed very few “sticky” codons in frame 2 and displayed constant ribosome occupancy along the coding region (Figure 6(b)). Interestingly, the distribution of “sticky” codons among the reading frames of* OAZ1* is not uniform (Figure 6(c)). The stop codon of* OAZ1*, serving as the +1 PRF site, appears to be a dividing point for “sticky” codon composition in frame 2. Since frame 2 becomes frame 1 after +1 PRF, this result supports the view that codon optimization is highly coordinated between general coding regions and specific PRF signals.

## 3. Discussion

Maintaining reading frame fidelity during mRNA translation is a cellular imperative. In general, frameshift errors are more deleterious than missense errors in protein production. While amino acid mis-incorporation is primarily monitored in the decoding center via sampling tRNAs, mechanisms controlling the step size of translocation are poorly understood. The accuracy of decoding itself, however, is insufficient to account for the high degree of reading frame fidelity in translation of long messages. For the ribosome to adhere to the correct reading frame during the entire course of translation, a retrospective fidelity check by the ribosome after translocation seems to be critical. Interestingly, in bacteria, the internal SD:anti-SD interaction is able to reposition the ribosome from frame 0 to frame 1, thereby causing +1 PRF. This feature is well documented in the* prfB* gene and is essential for the synthesis of full length release factor 2 (RF2) [41, 42]. Although other factors are essential in this PRF, this phenomenon suggests that mRNA:rRNA pairing plays a role in controlling translational reading frames. Analogous to prokaryotic SD:anti-SD duplex formation, eukaryotic mRNA:rRNA pairing has been reported to play a role in noncanonical translation initiation [43, 44], as well as reinitiation [45]. However, the physiological significance of mRNA:rRNA interaction during elongation remained elusive.

We found that the feature of mRNA:rRNA pairing plays a similar role in eukaryotic translation. Importantly, it provides a postdecoding “gripping” mechanism to prevent overmovement of mRNA during translocation. This notion is supported by several lines of evidence. First, the FS reporter assay showed greater than 4-fold higher frameshift errors when the mRNA:rRNA interaction is disrupted by replacing the “sticky” codons with the “non-sticky” sequence. Second, we observed a high frequency of –1 FS when the “sticky” sequence was relocated from frame 0 to frame 2. Third, codon composition analysis revealed that “sticky” codons are generally avoided in frame 2 for the majority of genes, suggesting a strong selection for codon optimization. Fourth, for genes experiencing PRF (such as* OAZ1*), the “sticky” codons are clearly redistributed among different reading frames. Although the functional purpose of individual cases may differ, the advantage associated with mRNA:rRNA pairing offers a clear mechanistic view of reading frame maintenance during translation.

For the ribosome to achieve translational accuracy, it is conceivable that at least two types of RNA interaction occur during the course of translation: mRNA:tRNA pairing at the decoding center and mRNA:rRNA pairing at the exit tunnel of the mRNA path. While the former ensures proper mRNA decoding via tRNA sampling, the latter secures the correct reading frame via a gripping mechanism. Unlike the continuous codon:anti-codon pairing, the appearance of “sticky” codons along the primary reading frame is periodic because the majority of 61 sense codons are “non-sticky” (Figure 6(a)). How does the periodic mRNA:rRNA interaction contribute to continuous reading frame precision? We reasoned that the frame-specific effect of “sticky” codons offers an autocorrecting mechanism to achieve retrospective fidelity control. For instance, once the “non-sticky” codons reach the exit site of the mRNA path, the lack of mRNA grip may cause a higher chance of +1 FS (Figure 4(a)). However, the recurrence of the next “sticky” codons in frame 0 (now becomes frame 2 after +1 FS) has the potential to induce –1 FS (Figure 4(b)), which ultimately cancels the original +1 FS. Therefore, the periodic presence of “sticky” codons is able to maintain reading frame accuracy via self-guard and autocorrection. The remarkable reading frame fidelity is well reflected by the fact that the majority of transcripts have minimal ribosome drop-off during translation (Figure 1(c)).

It is intriguing that the eukaryotic mRNA:rRNA pairing is centered on AUG, a codon essentially selected for initiation. For any given transcript, it is the position of the start codon that defines the corresponding open reading frame. Despite the codon degeneracy, it is puzzling that the start codon AUG is shared with the internal methionine codon. Since the AUG codon is complementary to the CAU triplet at the 3′ end of 18S rRNA, it is tempting to speculate that its “stickiness” helps retain the translation machinery in the reading frame originally set by AUG. Robustness to frameshift errors is likely an inherent constraint on the early genetic code. As a result, selection of AUG as a start codon lowers the fitness cost by minimizing frameshifting errors. Notably, recent development of initiating ribosome profiling in eukaryotes revealed prevalence of non-AUG start codons in eukaryotes [32, 46, 47]. Notably, the majority of those non-canonical initiators belong to AUG-like codons, which bear similar “stickiness” as AUG in mediating mRNA:rRNA pairing. Additionally, the highly conserved 3′ end of 18S rRNA has been found to be heavily modified in both precursor and mature forms [48]. The critical role of differential nucleotide modification in mRNA:rRNA interaction awaits further investigation. It is possible that the ribosome serves as a molecular selector by employing mRNA:rRNA interaction to shape the evolution of the start codon as well as codon optimization in the coding region.

A growing body of evidence indicates that DNA sequences that code for proteins convey more information than the protein-coding instruction. Many “parallel codes” embedded within nucleotide sequences include signals for RNA splicing, modification, localization, secondary structure formation, and functional regulation [49–51]. In this study, we uncovered a hidden principle from protein-coding sequences that governs ribosome dynamics and reading frame fidelity during translation.

## 4. Materials and Methods

### 4.1. Cells and Reagents

HEK293 and MEF cells were maintained in Dulbecco's Modified Eagle's Medium (DMEM) with 10% fetal bovine serum (FBS). Cycloheximide (CHX) was purchased from Sigma. Plasmids transfection was performed using Lipofectamine 2000 (Invitrogen) following the manufacturer's instruction.

### 4.2. Plasmids

CrPV bicistronic reporter construct containing CrPV IRES was kindly provided by Sunnie Thompson (University of Alabama). To construct non-AUG CrPV reporter,* Renilla* gene was amplified with a primer set of Renilla-F/Renilla-R and cloned into* Hind*III/*Xho*I sites of CrPV plasmid. To construct Flag-Renilla CrPV reporter, Flag oligos were annealed and cloned to Hind III/BamH I sites of non-AUG CrPV plasmid. To construct frameshifting reporters, synthesized sense and anti-sense DNA oligos (see Table 1) were annealed and cloned to* BamH*I/*EcoR*I sites of Flag-Renilla CrPV plasmid. The plasmid expressing 18S rRNA was kindly provided by Vincent P. Mauro (The Scripps Research Institute). For 18S rRNA mutant, mutagenesis was performed using the site-directed mutagenesis kit following the manufacturer's instruction (New England Biolabs). Sequences of all the primers used for plasmid construction are listed in Supplemental Primer Table. All plasmids were confirmed by DNA sequencing.

### 4.3. Dual-Luciferase Assay

Cells were transfected with each luciferase construct and luciferase activity was measured 24 hours after transfection by the Dual-Luciferase assay system (Promega). The Renilla luciferase activity was normalized to Firefly luciferase. For anti-Flag immunoprecipitation of Fluc, whole cell lysates were incubated with anti-Flag M2 magnetic beads (Sigma-Aldrich). After 3 times of wash with phosphor-buffer saline (PBS), the beads were directly mixed with Fluc substrates (Promega) on a 96-well plate followed by luminometer detection.

### 4.4. *In Vitro* Transcription

Plasmids containing various Rluc reporters were used as templates. Transcripts with normal m^7^G cap were generated using the mMessage mMachine T7 Ultra kit (Ambion). All mRNA products were purified using the MEGAclear kit (Ambion) according to the manufacturer's instruction.

### 4.5. Polysome Profiling

Cells were first treated with cycloheximide (100 *μ*g/mL) for 3 min at 37°C to immobilize the translating ribosomes. After ice-cold PBS solution wash, cells were then harvested by ice-cold polysome lysis buffer [10 mM Hepes, pH 7.4, 100 mM KCl, 5 mM MgCl_2_, 100 *μ*g/mL cycloheximide, and 2% (vol/vol) Triton X-100]. After centrifugation at 12,000 × g for 10 min at 4°C, the supernatant was subjected to sucrose gradient sedimentation. Briefly, sucrose solutions were prepared in polysome gradient buffer (10 mM Hepes, pH 7.4, 100 mM KCl, 5 mM MgCl_2_, 100 *μ*g/mL cycloheximide). Sucrose density gradients [15–45% (wt/vol)] were freshly made in SW41 ultracentrifuge tubes (Fisher) using a BioComp Gradient Master (BioComp) according to the manufacturer's instructions. Approximately 650 *μ*L of cell lysate was loaded onto sucrose gradients, followed by centrifugation for 100 min at 38,000 rpm, 4°C, in an SW41 rotor. Separated samples were fractionated at 0.375 mL/min by using a fractionation system (Isco) that continually monitored OD254 values. Fractions were collected into tubes at 1-min intervals.

### 4.6. cDNA Library Construction of Ribosome-Protected mRNA Fragments

To convert the polysome into monosome,* E. coli *RNase I (Ambion) was added to the pooled polysome samples (750 U per 100 A260 units) and incubated for 1 h at 4°C. Total RNA extraction was performed using TRIzol reagent. Purified RNA samples were dephosphorylated in a 15 *μ*L reaction containing 1× T4 polynucleotide kinase buffer, 10 U SUPERase_In, and 20 U T4 polynucleotide kinase (NEB). Dephosphorylation was carried out for 1 h at 37°C, and the enzyme was then heat inactivated for 20 min at 65°C. Dephosphorylated samples were then mixed with 2× Novex TBE-urea sample buffer (Invitrogen) and loaded on a Novex denaturing 15% polyacrylamide TBE-urea gel (Invitrogen). The gel was stained with SYBR Gold (Invitrogen) to visualize the RNA fragments. Gel bands containing RNA species corresponding to 28 nt were excised and physically disrupted by using centrifugation through the holes of the tube. RNA fragments were dissolved by soaking overnight in gel elution buffer (300 mM NaOAc, pH 5.5, 1 mM EDTA, 0.1 U/*μ*L SUPERase_In). The gel debris was removed using a Spin-X column (Corning) and RNA was purified by using ethanol precipitation. Purified RNA fragments were resuspended in 10 mM Tris (pH 8) and denatured briefly at 65°C for 30 s. Poly-(A) tailing reaction was performed in an 8 *μ*L with 1 × poly-(A) polymerase buffer, 1 mM ATP, 0.75 U/*μ*L SUPERase_ In, and 3 U* E. coli* poly-(A) polymerase (NEB). Tailing was carried out for 45 min at 37°C. For reverse transcription, the following oligos containing barcodes were synthesized:

MCA02, 5′-pCAGATCGTCGGACTGTAGAACTCTØCAAGCAGAAGACGGCATACGATT  TTTTTTTTTTTTTTTTTTVN-3′;

LGT03, 5′-pGTGATCGTCGGACTGTAGAACTCTØCAAGCAGAAGACGGCATACGATT  TTTTTTTTTTTTTTTTTTVN-3′;

YAG04, 5′-pAGGATCGTCGGACTGTAGAACTCTØCAAGCAGAAGACGGCATACGATT  TTTTTTTTTTTTTTTTTTVN-3′;

HTC05, 5′-pTCGATCGTCGGACTGTAGAACTCTØCAAGCAGAAGACGGCATACGATT  TTTTTTTTTTTTTTTTTTVN-3′.

In brief, the tailed RNA product was mixed with 0.5 mM dNTP and 2.5 mM synthesized primer and incubated at 65°C for 5 min, followed by incubation on ice for 5 min. The reaction mix was then added with 20 mM Tris (pH 8.4), 50 mM KCl, 5 mM MgCl_2_, 10 mM DTT, 40 U RNaseOUT, and 200 U SuperScript III (Invitrogen). RT reaction was performed according to the manufacturer's instructions. Reverse transcription products were separated on a 10% polyacrylamide TBE-urea gel as described earlier. The extended first-strand product band was expected to be approximately 100 nt, and the corresponding region was excised. The cDNA was recovered by using DNA gel elution buffer (300 mM NaCl, 1 mM EDTA). First-strand cDNA was circularized in 20 *μ*L of reaction containing 1× CircLigase buffer, 2.5 mMMnCl_2_, 1 M Betaine, and 100 U CircLigase II (Epicentre). Circularization was performed at 60°C for 1 h, and the reaction was heat inactivated at 80°C for 10 min. Circular single-strand DNA was relinearized with 20 mM Tris-acetate, 50 mM potassium acetate, 10 mM magnesium acetate, 1 mM DTT, and 7.5 U APE 1 (NEB). The reaction was carried out at 37°C for 1 h. The linearized single-strand DNA was separated on a Novex 10% polyacrylamide TBE-urea gel (Invitrogen) as described earlier. The expected 100-nt product bands were excised and recovered as described earlier [33].

### 4.7. Deep Sequencing

Single-stranded template was amplified by PCR by using the Phusion High-Fidelity enzyme (NEB) according to the manufacturer's instructions. The oligonucleotide primers qNTI200 (5′-CAAGCAGAAGACGGCATA- 3′) and qNTI201 (5′-AATGATACGGCGACCACCG ACAGGTTCAGAGTTCTACAGTCCGACG- 3′) were used to create DNA suitable for sequencing, i.e., DNA with Illumina cluster generation sequences on each end and a sequencing primer binding site. The PCR contains 1× HF buffer, 0.2 mM dNTP, 0.5 *μ*M oligonucleotide primers, and 0.5 U Phusion polymerase. PCR was carried out with an initial 30 s denaturation at 98°C, followed by 12 cycles of 10 s denaturation at 98°C, 20 s annealing at 60°C, and 10 s extension at 72°C. PCR products were separated on a nondenaturing 8% polyacrylamide TBE gel as described earlier. Expected DNA at 120 bp was excised and recovered as described earlier. After quantification by Agilent BioAnalyzer DNA 1000 assay, equal amount of barcoded samples was pooled into one lane. Approximately 3–5 pM mixed DNA samples were used for cluster generation followed by sequencing using sequencing primer 5′-CGACAGGTTCAGAGTTC TACAGTCCGACGATC-3′ (Illumina HiSeq).

### 4.8. Estimating Remaining Ribosomes on mRNA

Upon –1 or +1 frameshift, frequent stop codons lead to early translation termination and ribosome dissociation. The amount of remaining ribosomes on the transcript is a combination of ribosomes in the reading frames 0, 1, and 2. For example, the amount of ribosome in frame 0 of the ith codon is attributed to (a) an in-frame 3nt movement from frame 0 of the (i-1)th codon; (b) a -1 frameshift movement (2nt) from frame 1 of the (i-1)th codon; and (c) a +1 frameshift movement (4nt) from frame 2 of the (i-2)th codon. Therefore, at each nucleotide position of mRNA, the percentage of remaining ribosome can be estimated by following equations with the initial amount of ribosomes at the start codon as 100%

(1) Ri,0=p0Ri−1,0+p−1Ri−1,1+p+1Ri−2,2,if  i>2p0Ri−1,0,if  i=21,if  i=1Ri,1=p0Ri−1,1+p+1Ri−1,0+p−1Ri−1,2,if  i>10,if  i=1  or  stop  codonRi,2=p0Ri−1,2+p−1Ri,0+p+1Ri−1,1,if  i>10,if  i=1  or  stop  codon

*R*(*i*, 0), *R*(*i*, 1), and *R*(*i*, 2) are percentages of ribosomes at codon *i* for the reading frames 0, 1, and 2, respectively. *p*_−1_and *p*_+1_are the probabilities of ribosome making –1 and +1 frameshift with the fixed values of 0.1% and 0.02% according to the Luciferase Assay, while *p*_0_is the probability of ribosome staying in the same reading frame, which is 99.88%.

### 4.9. Compiling Longest-Isoform Transcriptome Datasets

By default, all the computational analyses are conducted on transcriptome datasets consisting of longest mRNA transcripts according to the NCBI RefSeq annotation. Generally, mRNA isoform of largest CDS length is defined as the longest isoform of the gene. In the case of equal CDS lengths between different isoforms, 5′ UTR and 3′ UTR are compared orderly.

### 4.10. Processing Ribo-Seq Reads

Tophat was used to map Ribo-Seq reads (25nt to 35nt) to transcriptome and genome with parameters (--bowtie1 –p 10 –no-novel-juncs) [52]. The 1st, 4th, 7th, 10th, 13th, 16th, 19th, 22nd, and 25th nucleotide of uniquely mapped RPF are mapped to NCBI RefSeq genes to construct the position-specific Ribo-Seq profile for the downstream analyses. For example, to analyze the average in-frame rate of 5′ end codon of RPF, the expression profile of 1st nucleotide of uniquely mapped RPF was used.

### 4.11. Calculating Average In-Frame Rate (*IFR*)

A two-step procedure is employed to calculate *IFR* of Ribo-Seq experiments. First, in each individual gene, an *IFR* is calculated for each one of the 61 sense codons:

(2) $$\begin{array}{c} {IFR}_{\left(codon\;\;x,gene\;\;i\right)}=\frac{\sum_{m=1}^{k}F_{m0}}{\sum_{m=1}^{k}\left(F_{m0}+F_{m1}+F_{m2}\right)} \end{array}$$

*k* is the total number of *codon*  *x*, *F*_*m*0_, *F*_*m*1_  *and*  *F*_*m*2_ are the number of reads of the *m*_*th*_  *codon*  *x* in the reading frames 0, 1, and 2, respectively. Codons with less than 10 Ribo-Seq reads in the particular gene are disregarded for* IFR* calculation. Next, an average value is computed over all the qualified genes in the Ribo-Seq experiments to represent the global average *IFR* of *codon*  *x*.

### 4.12. Calculating Average Codon Coverage Score (ACCscore)

To calculate ACCscore, an internal gene normalization is first conducted by dividing the raw Ribo-Seq reads by the average number of reads per nucleotide of the CDS (total number of reads divided by the length of CDS). Next, for each of the 61 sense codons in the gene, an ACCscore is calculated as

(3) $$\begin{array}{c} {ACCscore}_{codon\;\;x,gene\;\;i}=\frac{\sum_{m=1}^{K}N_{m}}{K} \end{array}$$

*N*_*m*_ is the normalized value of the *m*_*th*_  *codon*  *x* of *gene*  *i* and K is the total number of *codon*  *x* of *gene*  *i*. Finally, a global ACC score for each of the 61 sense codons is calculated by averaging the ACC scores of individual genes.

### 4.13. Aligning Ribo-Seq Profile to the n_th_ Codon

The position of n_th_ codon of a particular gene is retrieved from NCBI RefSeq annotation. Raw Ribo-Seq densities were first normalized by the average density per nucleotide of the CDS. A window of 90nt (–30nt,+ 60nt) is selected for each gene to make an average aggregation plot over the whole transcriptome.

### 4.14. Codon Composition in Three Reading Frames of CDS

The total number of each sense codon in the annotated CDS (frame 0), frame 1, and frame 2 is counted for the longest mRNA isoform of individual genes. The codon fraction is calculated by dividing the count of a particular codon in a single reading frame (frame 0, 1, or 2) by the sum of codon frequencies in the three reading frames. Finally, average value of codon fraction is computed over all the protein-coding genes. The NCBI RefSeq gene annotations (human:hg19; mouse:mm10; chimpanzee:panTro4; Rhesus Macaque:rheMac3; dog: canFam3; cat:felCat5; chicken:galGal4; cow:bosTau7; zebrafish:danrer7) were downloaded from UCSC genome browser for the above analyses [53].

### 4.15. tRNA Adaptation Index

As described previously, tRNA adaptation indexes (tAI) are computed with codonR [54] using tRNA copy number data for human (hg19) and mouse (mm10) from GtRNAdb [55].

### 4.16. Codon Randomization Analysis

To eliminate the possibility that the minimal representation of ATG-like codons in frame 2 is not a by-product of frame-0 codon bias, the CDS sequences of the longest mRNA isoforms according to NCBI RefSeq annotation were retrieved for codon or dinucleotide randomization analysis. Specifically, frame-0 codons or the first two nucleotides of frame-0 codons of each individual mRNA were randomly shuffled. For each shuffled CDSome, average codon fraction values of 61 sense codons were calculated based on the method described in the “Codon Composition in Three Reading Frames of CDS”. This random process is repeated for 100 times. As a result, a comparison was made to measure the difference between actual codon fraction in frame 2 to the distribution of random values.

## Figures and Tables

**Table 1:** Oligos.

Gene name	Primer name	Primer sequence
Non-AUG Renilla	Renilla-F	CCCAAGCTTCCACCATCGGGATCCCCACCATCGGAATTCacttcgaaagtttatgatc
	Renilla-R	CCCTCGAGcccctagaattattgttc

Flag frame 0	Flag-0-S	AGCTTGCCACCATGgattacaaggacgacgacgataagG
	Flag-0-AS	GATCCcttatcgtcgtcgtccttgtaatcCATGGTGGCA

+1 FS	FS1-S	GATCCACGATCAAGCTGTTGGTGCTGCG
	FS1-AS	AATTCGCAGCACCAACAGCTTGATCGTG

−1 FS	FS2-S	GATCCACGATCAAGCTGTTGGTGCTGCAG
	FS2-AS	AATTCTGCAGCACCAACAGCTTGATCGTG

Non-sticky frame 0	NS-0-S	GATCCGGAGACGGCGGAGGTGGAGACG
	NS-0-AS	AATTCGTCTCCACCTCCGCCGTCTCCG

Non-sticky FS1	NS-FS1-S	GATCCGGAGACGGCGGAGGTGGAGACCG
	NS-FS1-AS	AATTCGGTCTCCACCTCCGCCGTCTCCG

Non-sticky FS2	NS-FS2-S	GATCCGGAGACGGCGGAGGTGGAGACCAG
	NS-FS2-AS	AATTCTGGTCTCCACCTCCGCCGTCTCCG

+1 FS frame 1	+1 FS-S	GATCCCACGATCAAGCTGTTGGTGCTGG
	+1 FS-AS	AATTCCAGCACCAACAGCTTGATCGTGG

−1 FS frame 2	+1 FS-S	GATCCCAACGATCAAGCTGTTGGTGCTGG
	+1 FS-AS	AATTCCAGCACCAACAGCTTGATCGTTGG

## Supplementary Materials

Supplementary Materials Supplemental information includes Supplemental Experimental Procedures, six figures, and three tables and can be found with this article online.
