# Supplementary material for: A Coding Sequence-Embedded Principle Governs Translational Reading Frame Fidelity
Source: Research (Wash D C). 2018 Sep 20;2018:7089174. doi: 10.1155/2018/7089174 (PMC6750092; doi:10.1155/2018/7089174)
Supplement: Supplementary Materials — Supplemental information includes Supplemental Experimental Procedures, six figures, and three tables and can be found with this article online. [file 7089174.f1.zip › 7089174.f1/Reading frame (SI) figure 020518.docx]

**
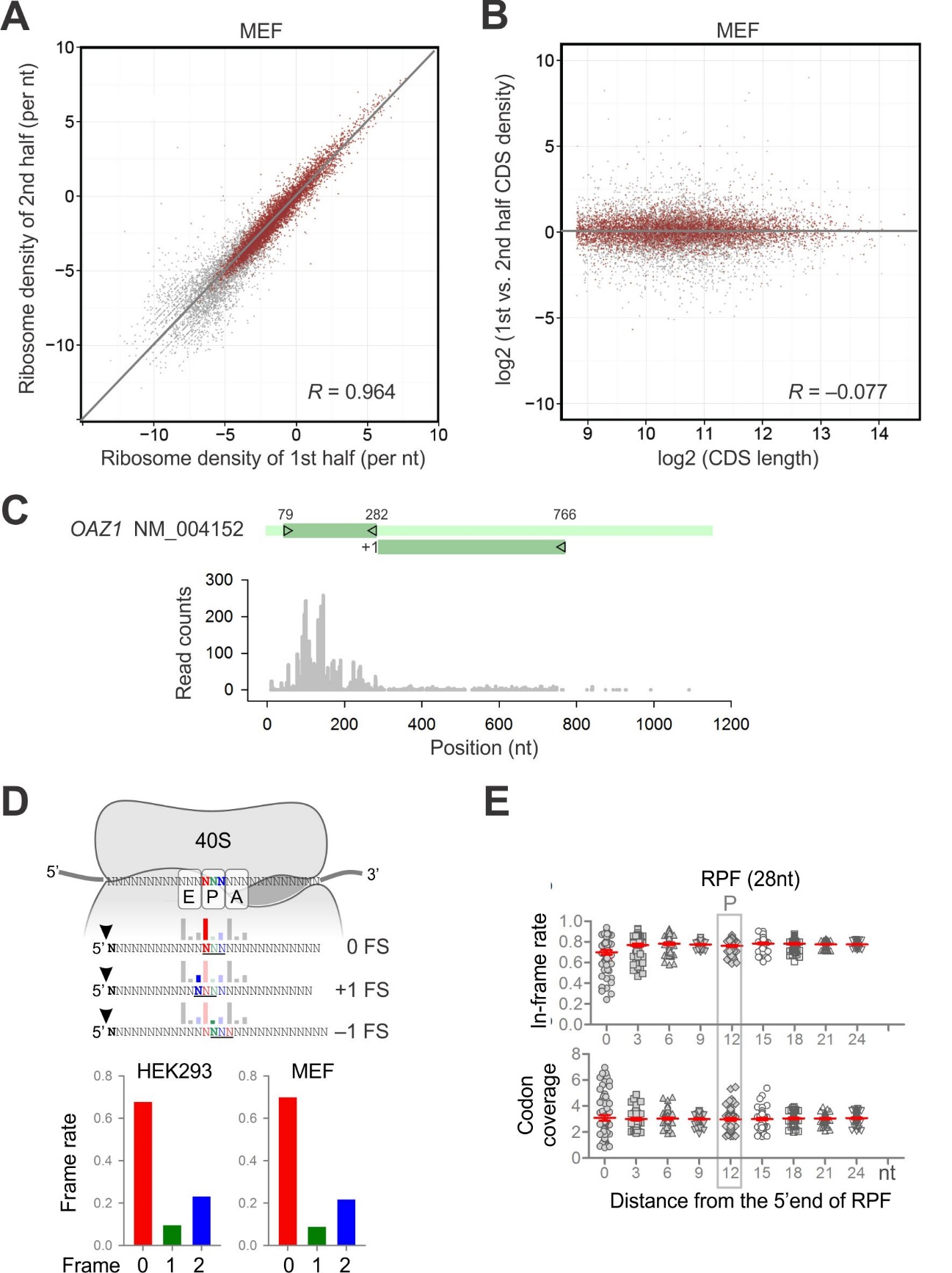
**

**Figure S1. Minimal ribosomal dropoff during translation**

1. Using ribosome profiling data in MEF cells, average ribosome density is plotted for the first and second half of each ORF. Genes with no less than 50 read counts in at least half of the CDS are shown in dark red.
2. Using ribosome profiling data in MEF cells, the ratio of ribosome density averaged between first and second half of each ORF is plotted as function of CDS length. Genes with no less than 50 read counts in at least half of the CDS are shown in dark red.
3. Examples of genes with decreased ribosome density in the second half of the coding region. Gene structure is shown above with coding regions presented as dark green in different reading frames. Ribosome density across the CDS is shown below.
4. The top panel depicts the relative positions of RPFs after +1 or –1 FS when the 5’-most nucleotide is used to infer the P-site position. The bottom panel shows percentage of total RPFs mapped to three reading frames.
5. RPFs with fixed length of 28 nt are stratified by the identity of codons at different positions of footprints. Their corresponding IFR values (middle panel) and abundance (bottom panel) are group plotted. Red line, mean ± SD.


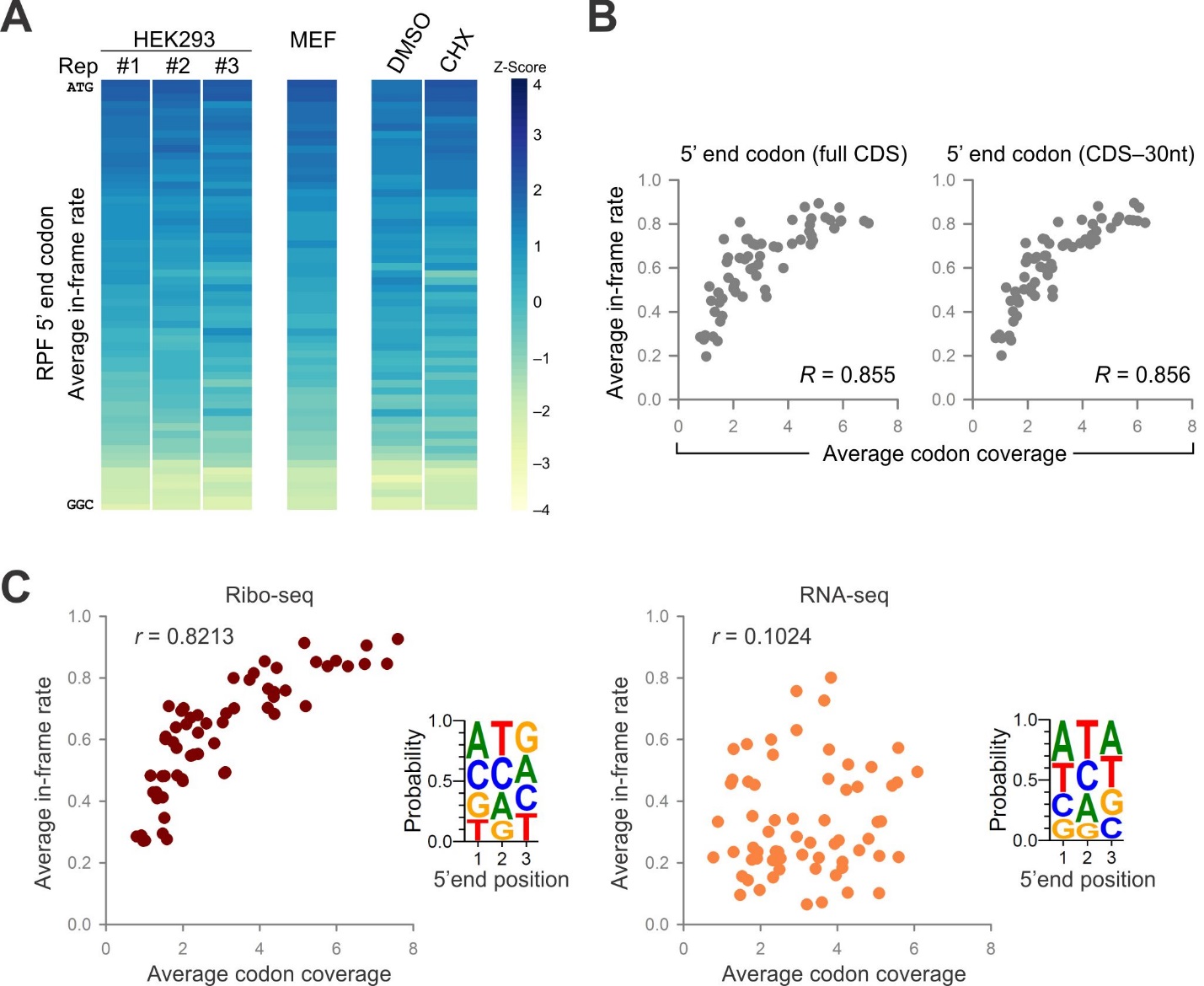


**Figure S2. The 5’ end codon preference of footprints is not due to technical bias**

1. Heatmap of average in-frame rate for RPFs with distinct 5’ end codons. Results from biological replicates, different cell lines, and different treatment are presented.
2. Using RPFs from HEK293 cells, all 61 sense codons are plotted by the average codon coverage score of RPFs with 5’end codons (x axis) against the average in-frame rate (y axis). The left panel uses RPFs mapped to the full CDS, whereas the right panel uses RPFs mapped to the CDS without counting the first 30 nt.
3. Using represented Ribo-seq and RNA-seq from MEF cells, all 61 sense codons are plotted by the average codon coverage score of RPFs with distinct 5’end codons (x axis) against the average in-frame rate (y axis). The inserted sequence logo shows the probability of individual nucleotides at 5’ end positions of RPFs.


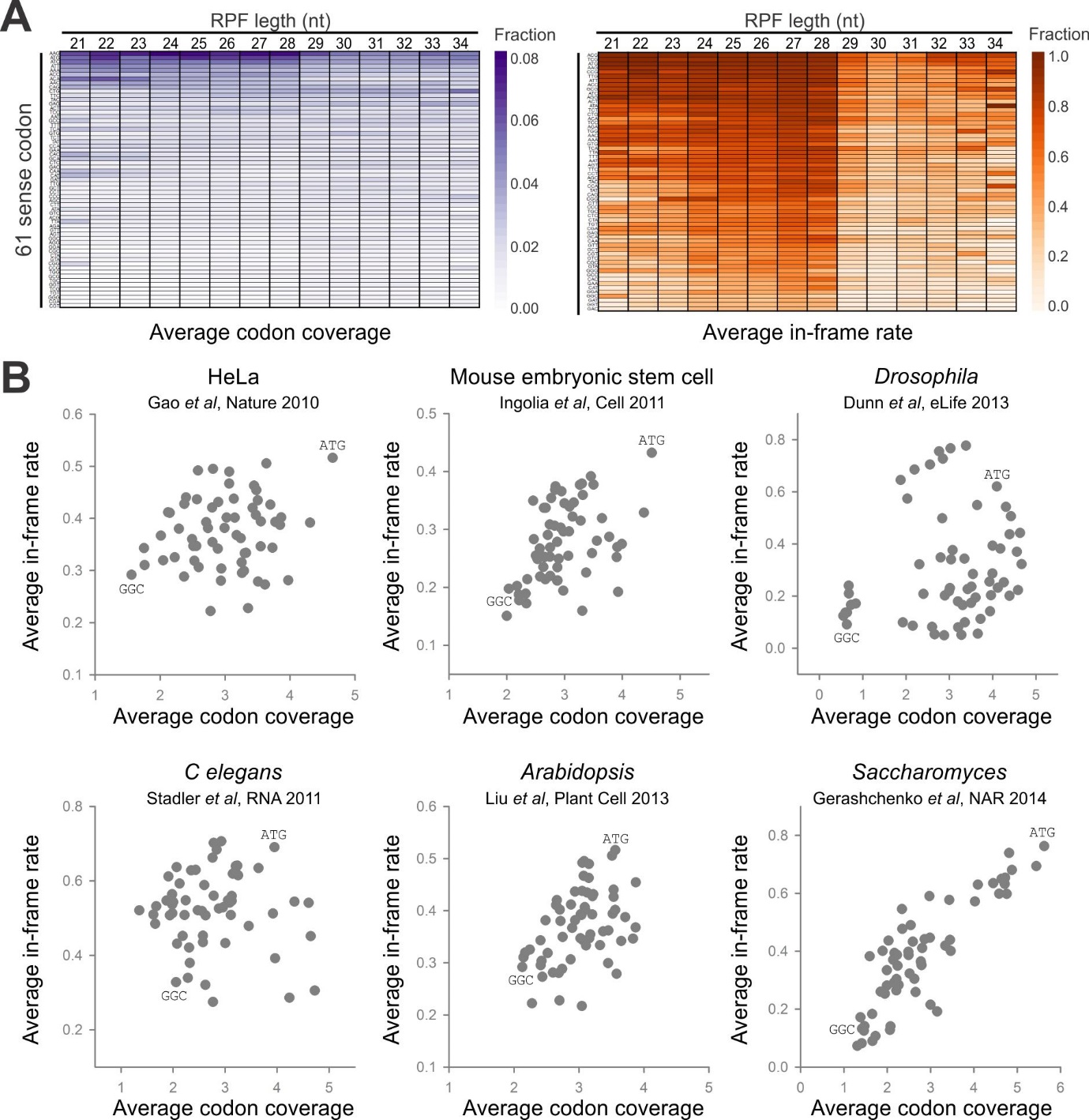


**Figure S3. The 5’ end codon identity of footprints influences read abundance and reading frame fidelity**

1. Using a representative Ribo-seq data set from HEK293 cells, RPFs with distinct 5’ end codon identity were stratified based on read length. Relative fractions of read abundance (left) and IFR (right) are presented in heatmap.
2. Correlation between average in-frame rate and codon coverage is shown using multiple data sets as indicated. Both ATG and GGC codons are highlighted.


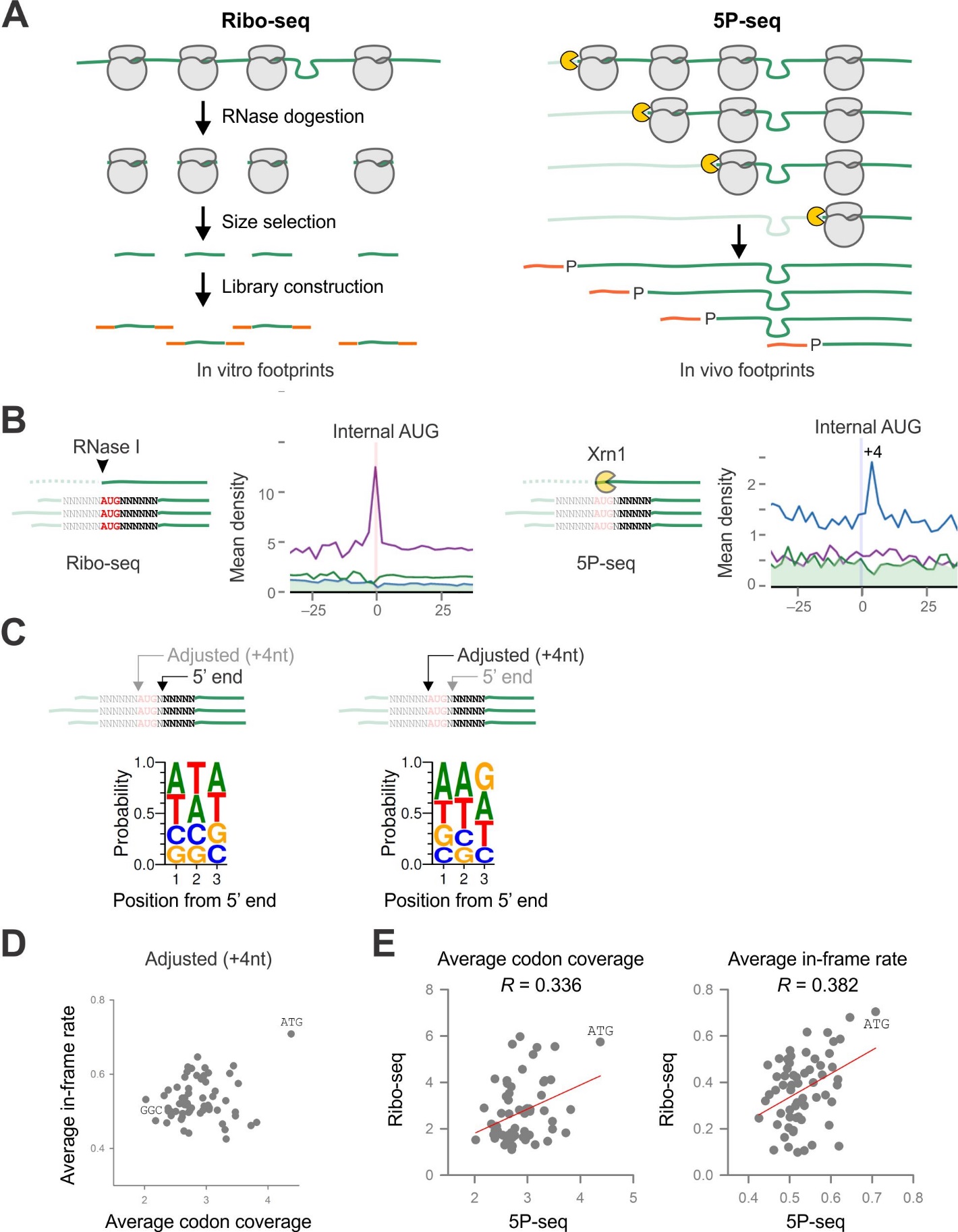


**Figure S4. 5’ end codon preference of *in vivo* ribosomal footprints derived from 5P-seq**

1. Experimental strategy of Ribo-seq and 5P-seq. While Ribo-seq captures ribosome footprints enriched in the lysates after nuclease digestion, 5P-seq captures footprints created in vivo during co-translational mRNA degradation (Pelechano, *et al*. Cell 161, 2015).
2. Metagene analysis of footprints obtained from Ribo-seq and 5P-seq. Transcripts are aligned at the second AUG codon of CDS. The average read density at each nucleotide position is plotted using 5’ end of reads. Different reading frames are color coded. Note the difference of peak position between Ribo-seq and 5P-seq.
3. Sequence logo shows the probability of individual nucleotides at 5’ end positions of reads.
4. After adjustment of 4 nt from the 5’ end of 5P-seq reads, correlation between average in-frame rate and codon coverage is shown as a scatter plot. Both ATG and GGC codons are highlighted.
5. Using 5P-seq and Ribo-seq (Gerashchenk *et al*, NAR 2014) data sets, all 61 sense codons are plotted by the average codon coverage score (left panel) and average in-frame rate (right panel). 5’end codons of RPFs are used for computing.


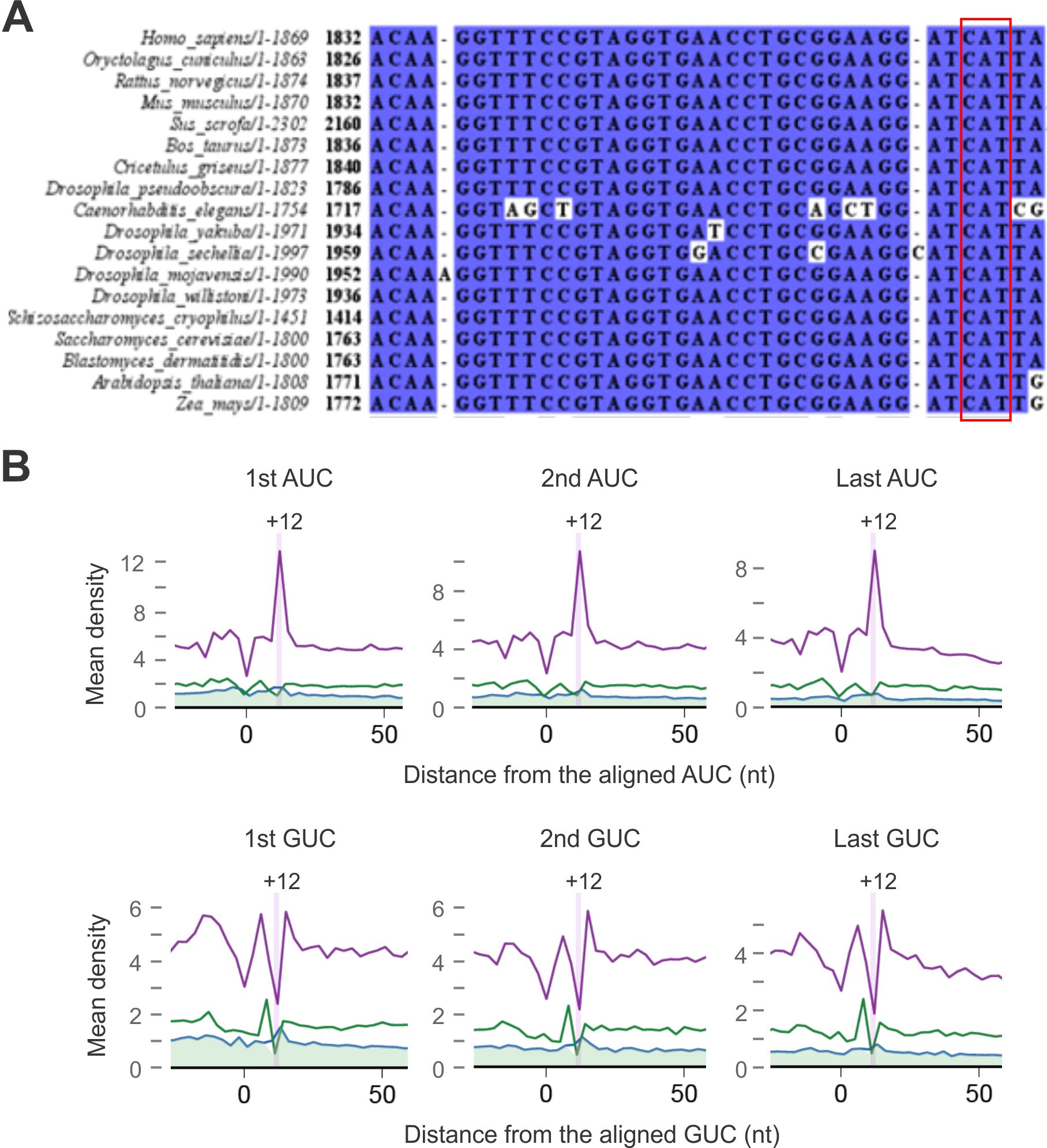


**Figure S5. mRNA:rRNA pairing leads to post-decoding translational pausing**

1. Multiple alignment of the 3’ end of 18S rRNA across eukaryotic species. The CAT triplet is highlighted in red.
2. Metagene analysis of RPFs obtained from HEK293 cells. Transcripts are aligned at different positions of AUC (left panel) or GUC (right panel). The average reads density at each nucleotide position is plotted using the P-site of RPFs. Mapped RPFs are stratified by reading frames (magenta, frame 0; blue, frame 1; green, frame 2). The +12 nt read peak is highlighted.


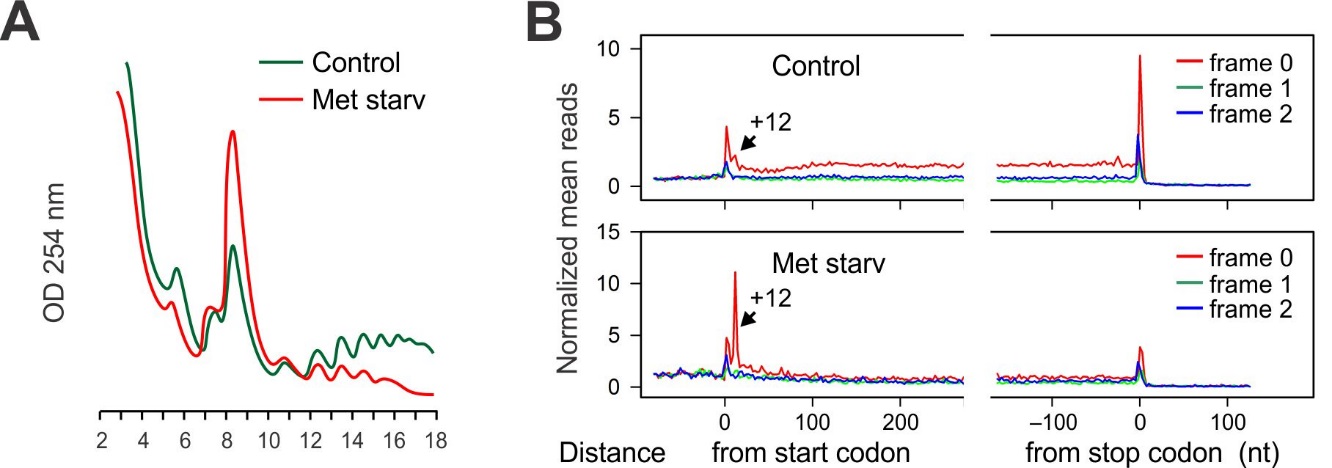


**Figure S6. Ribosome profiling of cells with methionine starvation.**

1. Sucrose gradient sedimentation of whole cell lysates from HEK293 cells with (red line) or without (green line) methionine starvation.
2. Metagene analysis of RPFs derived from HEK293 cells with (bottom panel) or without (top panel) methionine starvation. Normalized reads are averaged across the transcriptome, aligned at either their start or stop codons. Different reading frames are color coded.


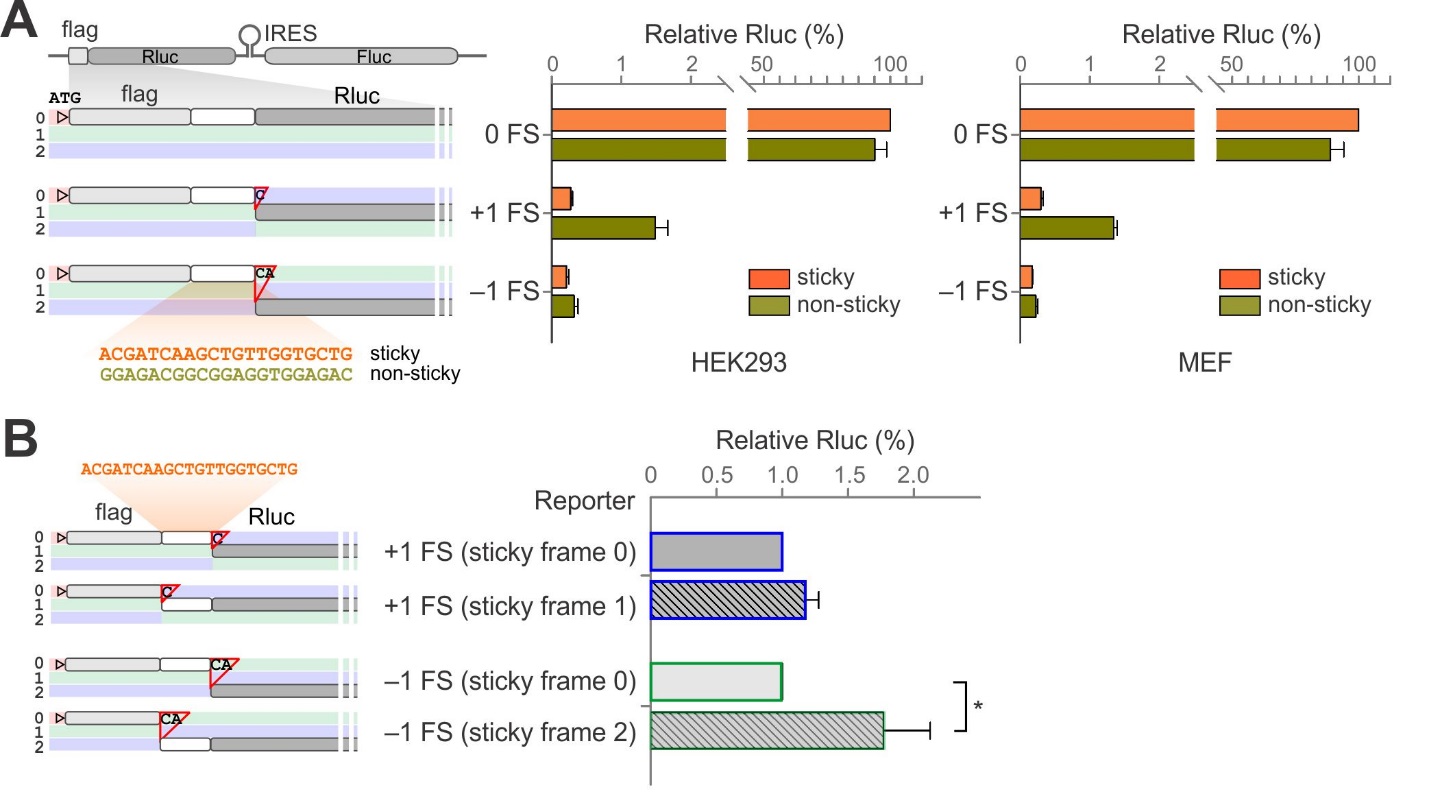


**Figure S7. mRNA:rRNA pairing exhibits reading frame-specific effects**

1. The left panel depicts schematic of FS reporters with “sticky” or “non-sticky” inserts in the frame 0 between flag and Rluc. The right panels show the relative Rluc activities in HeLa and MEF cells transfected with capped mRNA synthesized in vitro. Data are shown as means ± SEM, n = 3.
2. The left panel depicts schematic of FS reporters with “sticky” inserts in different frames between flag and Rluc. The right panels show the relative Rluc activities in MEF cells transfected with capped mRNA synthesized in vitro. Data are shown as means ± SEM, n = 3. * *P*<0.01, t-test.


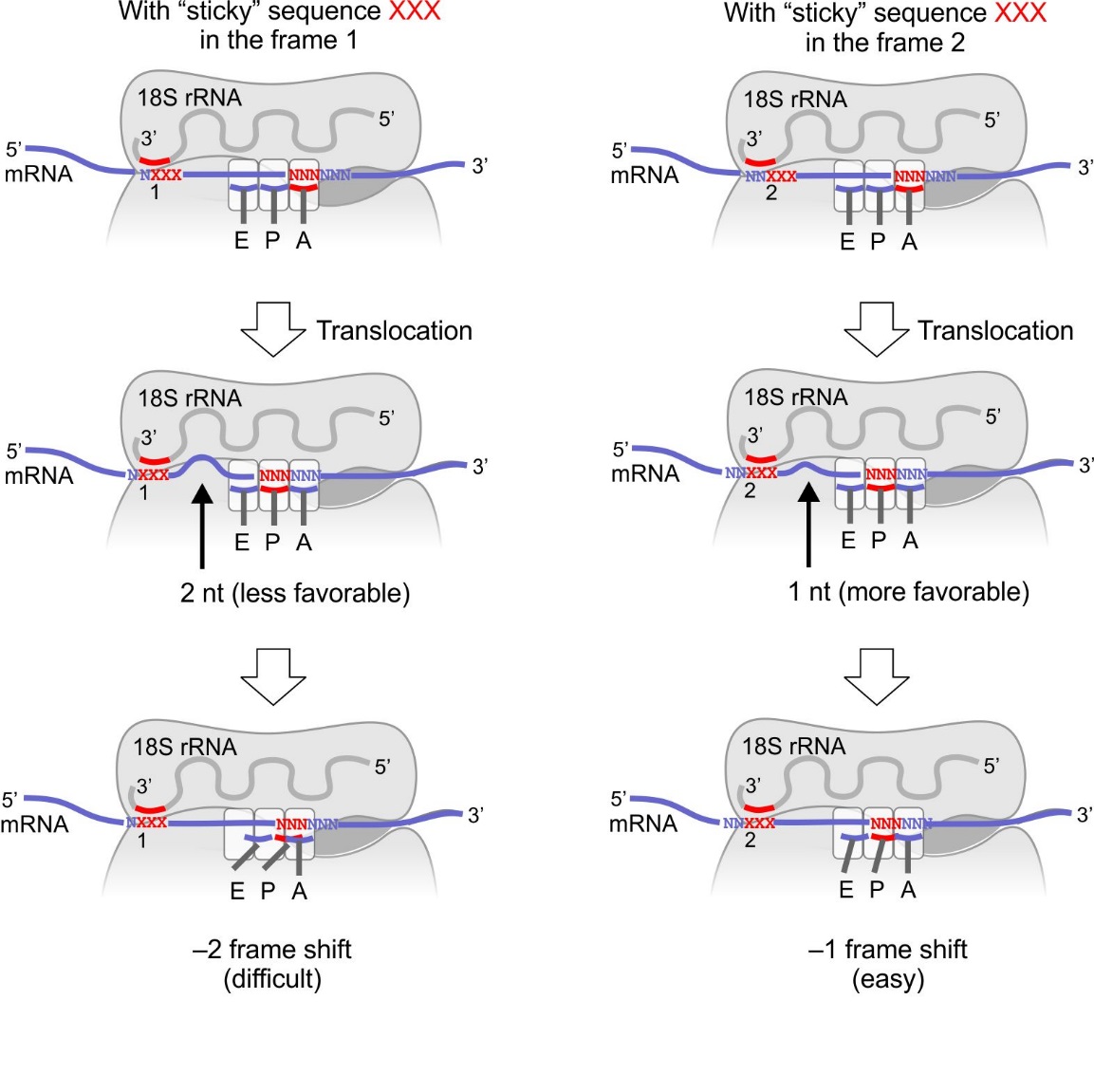


**Figure S8. Reading frame specific effects of “sticky” sequence in frameshifting**

Predicted frameshift errors when the “sticky” sequence is present in the frame 1 (left panel) or frame 2 (right panel). When the “sticky” sequence is present in the frame 1, mRNA:rRNA pairing is disfavored because it creates a stretched tension between the potential duplex and the codon: anticodon interaction in the decoding center. Upon translocation, the mRNA:rRNA interaction is still not favorable because it will lead to two extra nucleotides present in the spacer. In contrast, when the “sticky” sequence is located in the frame 2, mRNA:rRNA pairing is possible after translocation since only one extra nucleotide is present in the spacer. The counter force generated by the extra nucleotide is expected to drive 1 nt slipping back, resulting in –1 FS.

**
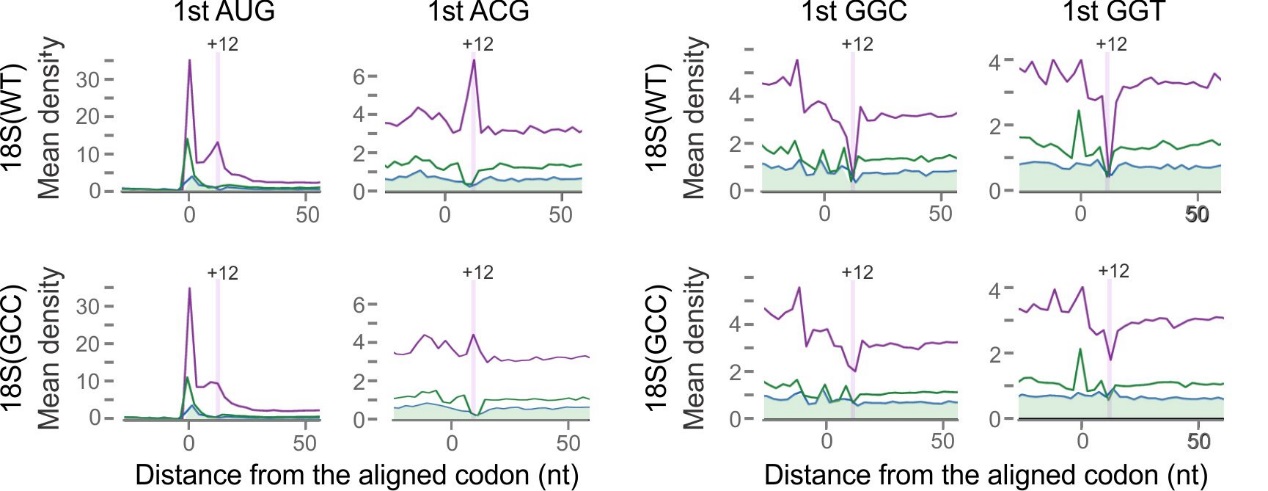
**

**Figure S9. Mutant 18S rRNA alters global codon “stickiness”**

Metagene analysis of RPFs obtained from HEK293 cells expressing wild type or mutant 18S rRNA. Transcripts are aligned at specific codons as indicated. The average read density at each nucleotide position is plotted using the P-site of RPFs. Mapped RPFs are stratified by reading frames (magenta, frame 0; blue, frame 1; green, frame 2). The +12 nt read peak is highlighted.


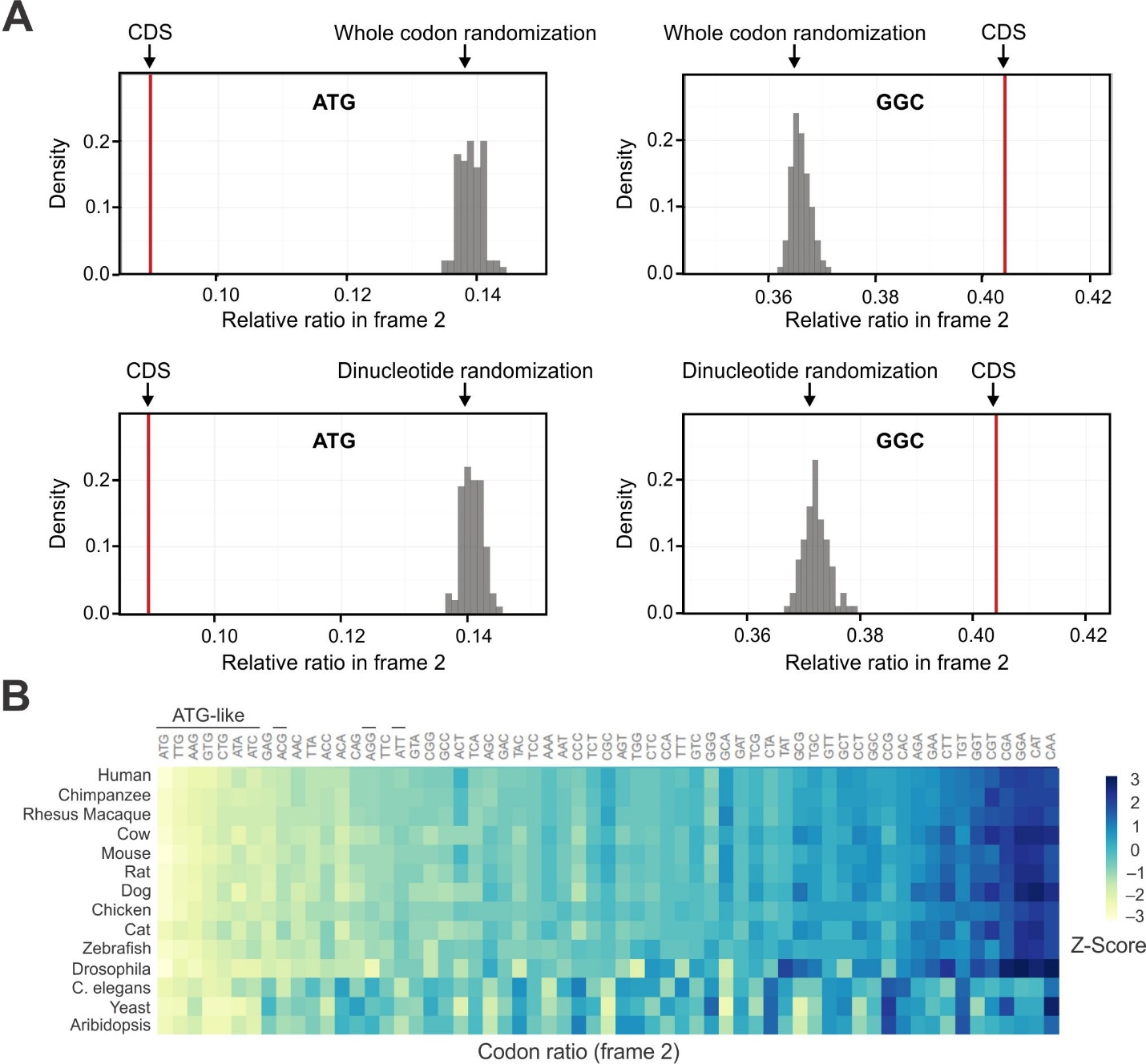


**Figure S10. “Sticky” codons exhibit frame-specific features.**

1. Human genome sequence (hg19) is used to perform dinucleotide randomization. The relative frequency of ATG and GGC in frame 2 is presented as histograms. Red line indicates the codon frequency derived from hg19.
2. Relative ratio of 61 sense codons in the frame 2 of different eukaryotic genomes is presented as a heat-map.
